# Increased macular choroidal blood flow velocity and decreased choroidal thickness with regression of punctate inner choroidopathy

**DOI:** 10.1186/1471-2415-14-73

**Published:** 2014-05-28

**Authors:** Kiriko Hirooka, Wataru Saito, Yuki Hashimoto, Michiyuki Saito, Susumu Ishida

**Affiliations:** 1 Department of Ophthalmology, Hokkaido University Graduate School of Medicine, Sapporo, Japan; 2 Department of Ocular Circulation and Metabolism, Hokkaido University Graduate School of Medicine, Nishi 7, Kita 15, Kita-ku, Sapporo 060-8638, Japan

**Keywords:** Choroidal blood flow velocity, Choroidal thickness, Enhanced depth imaging optical coherence tomography, Laser speckle flowgraphy, Punctate inner choroidopathy

## Abstract

**Background:**

Changes in choroidal circulation hemodynamics during the course of punctate inner choroidopathy (PIC) remain unknown. The aim of this study was to quantitatively evaluate changes in choroidal blood flow velocity by using laser speckle flowgraphy (LSFG) in patients with PIC.

**Case presentation:**

This PIC patient was initially treated with systemic corticosteroids for 4 months. LSFG measurements were taken 10 consecutive times before treatment and at 1, 3, 12, 20 and 23 months after the initiation of therapy. The mean blur rate (MBR), a quantitative index of relative blood flow velocity, was calculated using LSFG in three regions: Circles 1, 2 and 3 were set at the fovea, a lesion site, and an area of normal-appearing retina, respectively.

The PIC lesions scarred after treatment along with improvements in visual function and outer retinal morphology. When the changing rate of macular flow over the 12-month follow-up period was compared with the MBR before treatment (100%), an increase of 16–37%, 24–49% and 15–18% was detected in Circles 1, 2 and 3, respectively. At the time of PIC recurrence after 20 months, the MBR decreased temporarily but subsequently increased after retreatment with systemic corticosteroids. This trend was accompanied by a decrease in choroidal thickness at the lesion site after retreatment.

**Conclusions:**

Macular choroidal blood flow velocity increased and choroidal thickness decreased concurrently with regression of PIC. The present findings suggest that inflammation-related impairments in choroidal circulation may relate to the pathogenesis of PIC, extending over a wider area in the posterior pole than the PIC lesions per se.

## Background

Punctate inner choroidopathy (PIC) is an idiopathic chorioretinitis with a predilection for young myopic women. The condition is characterized by multiple punctate yellow-white chorioretinal lesions that present mainly in the posterior pole of the retina [1-4].

The pathogenesis of PIC is still largely unresolved. Indocyanine green angiography (ICGA) images obtained during the initial phase show hypofluorescence corresponding to the PIC lesions, suggesting inflammation-related choroidal circulation impairment [4,5]. The details of changes in choroidal circulation hemodynamics during the course of the disease remain unknown. Regarding the depth of the associated pathology, PIC lesions appear to affect the inner layer of the choroid, as indicated by the name of the disease [1]. These observations are based on previous studies using ICGA [5] and recent spectral-domain optical coherence tomography (SD-OCT) findings [6,7].

Laser speckle flowgraphy (LSFG) is a non-invasive tool that can be used to measure the blood flow velocity of ocular tissues including retinal and choroidal vessels without the administration of contrast agents [8]. The reproducibility of ocular blood velocity and flow measurements obtained using LSFG is high, and the time required for obtaining measurements is shorter than that required with the use of laser Doppler velocimetry [8,9]. The LSFG method is therefore suitable for monitoring changes in the ocular tissue circulation at the same sites at various intervals during the course of disease [10]. In the present study, we quantitatively measured choroidal blood flow velocity at the macula using LSFG in a PIC patient treated with systemic corticosteroids.

## Case presentation

**Figure 1:**
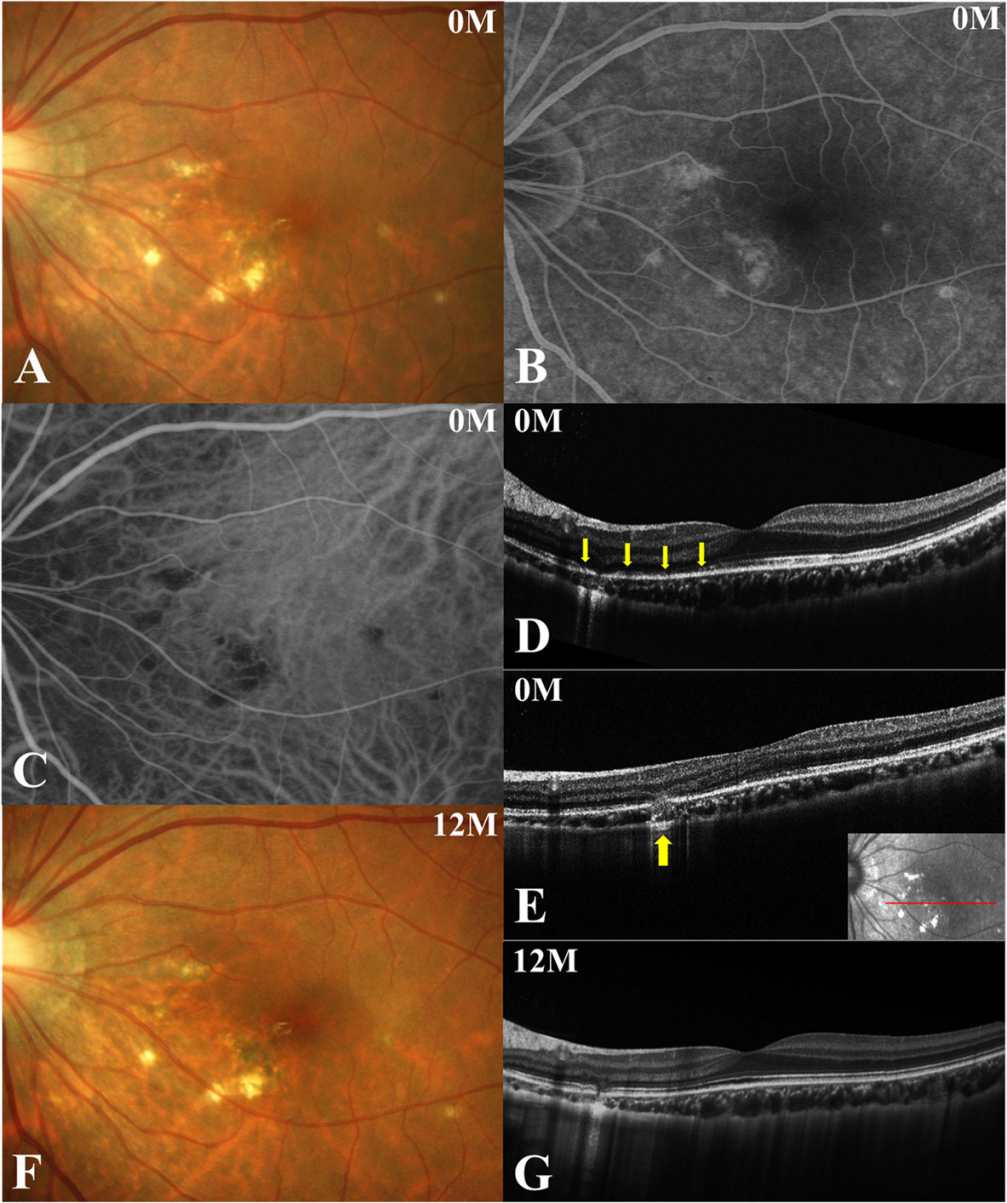
**Photographs of the left eye at the initial visit (A-E) and 12 months after the initial visit (F, G) in a patient with punctate inner choroidopathy (PIC). A**, Fundus photograph showing multiple punctate yellow-white exudates at the level of the retinal pigment epithelium in the posterior pole. **B**, Fluorescein angiography 600 second after dye injection showing hyperfluorescence corresponding to the exudates. **C**, The initial phase of indocyanine green angiography showing hypofluorescence corresponding to the lesions. **D**, **E**, Horizontal optical coherence tomography (OCT) image through the fovea shows the loss of photoreceptor inner/outer segment junction (IS/OS) integrity in the nasal area of the fovea (**D**, arrows), and exudate appearing as a massive, moderately reflective lesion extending from the outer nuclear layer to the choroid (**E**, arrow). **F**, The lesion was pigmented with some scarring. **G**, Horizontal cross-sectional OCT showed the recovery of IS/OS integrity.

**Figure 2:**
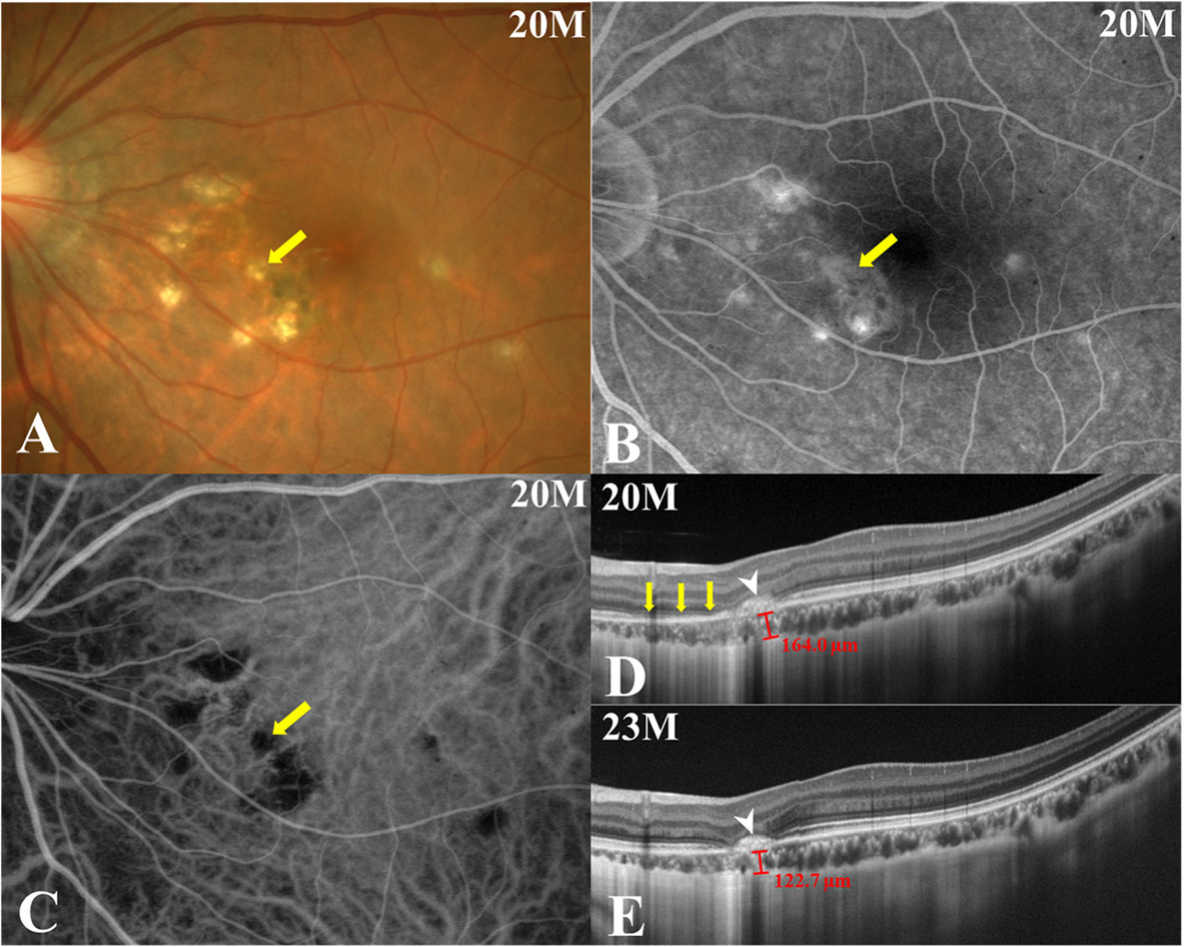
**Photographs of the left eye 20 months (at the time of PIC recurrence, A-D) and 23 months (E) after the initial visit. A**, The size of a punctate subretinal yellowish-white lesion (arrow) at the nasal side of the fovea OS had enlarged since the last visit (Figure 1A, F), although the number of PIC lesions remained unchanged. **B**, **C**, An area of hyperfluorescence on late-phase FA (**B**, arrow) or hypofluorescence on initial-phase ICGA (**C**, arrow) corresponding to the recurrent PIC lesion had expanded since the initial visit (Figure 1B, C). **D**, A cross-sectional enhanced-depth imaging (EDI)-OCT image taken at the same site depicted in Figure 1E showed the development of a hump-shaped hyperreflective chorioretinal nodule corresponding to the recurrent PIC lesion (arrowhead), with surrounding IS/OS loss (arrows). Choroidal thickness beneath the lesion was 164.0 μm. **E**, Three months after the resumption of systemic prednisolone therapy, the size of the hyperreflective lesion on EDI-OCT remained unchanged with slightly increased hyperreflectivity within the lesion (arrowhead) and improved IS/OS integrity. Choroidal thickness at the lesion site decreased to 122.7 μm.

A 19-year-old woman noticed blurred vision with photopsia of the left eye. The patient had no notable medical or family history. Her best-corrected visual acuity (BCVA) was 1.0 OD and 0.9 OS with myopia of −9.0 diopters OU. Visual examination of the right eye showed no abnormality. Slit-lamp biomicroscopy revealed mild cells in the anterior vitreous OS. Funduscopic examination revealed multiple punctate yellow-white exudates at the level of the retinal pigment epithelium (RPE) in the posterior pole but not the midperiphery OS (Figure 1A). These exudates appeared as initial hyperfluorescence with late staining on fluorescein angiography (FA, Figure 1B) and hypofluorescence during the initial phase of ICGA (Figure 1C). SD-OCT images showed a loss of photoreceptor inner/outer segment junction (IS/OS) integrity corresponding to the nasal fovea (Figure 1D), accompanied by a moderately reflective, nodule-like lesion extending from the outer nuclear layer to the choroid, which corresponded to the exudates described above (Figure 1E). Humphrey threshold 30–2 perimetry showed an area of decreased sensitivity corresponding to the lesion area. Multifocal electroretinography (mfERG) showed decreased amplitudes at the posterior pole wider than the lesion area OS and normal amplitudes OD. The patient received a diagnosis of PIC OS. A regimen of oral prednisolone (30 mg/day) was initiated then gradually tapered for 4 months. Three months after treatment, BCVA increased to 1.2 OS. The areas of exudate scarred as IS/OS line integrity recovered. Twelve months after treatment, some of the scar lesions developed hyperpigmentation (Figure 1F), whereas BCVA and OCT findings remained stable.Twenty months after the initial visit, the patient complained again of central blurred vision in the left eye. Her BCVA was 1.2 OS. A punctate subretinal yellowish-white lesion nasal to the fovea OS (Figure 1A, F) appeared to have increased in size (Figure 2A, arrow), although the number of PIC lesions remained unchanged. The area of hyperfluorescence on late-phase FA (Figure 2B, arrow) or hypofluorescence on initial-phase ICGA (Figure 2C, arrow) corresponding to the lesion had expanded since the patient’s initial visit (Figure 1B, C). The patient was diagnosed with recurrent PIC. Treatment with oral prednisolone (30 mg/day) was restarted and continued for 3 months with tapering. Three months after this second round of treatment, BCVA remained unchanged, and the recurrent lesion had scarred with an associated improvement of the patient’s subjective symptoms.

## Methods

### LSFG measurement

**Figure 3:**
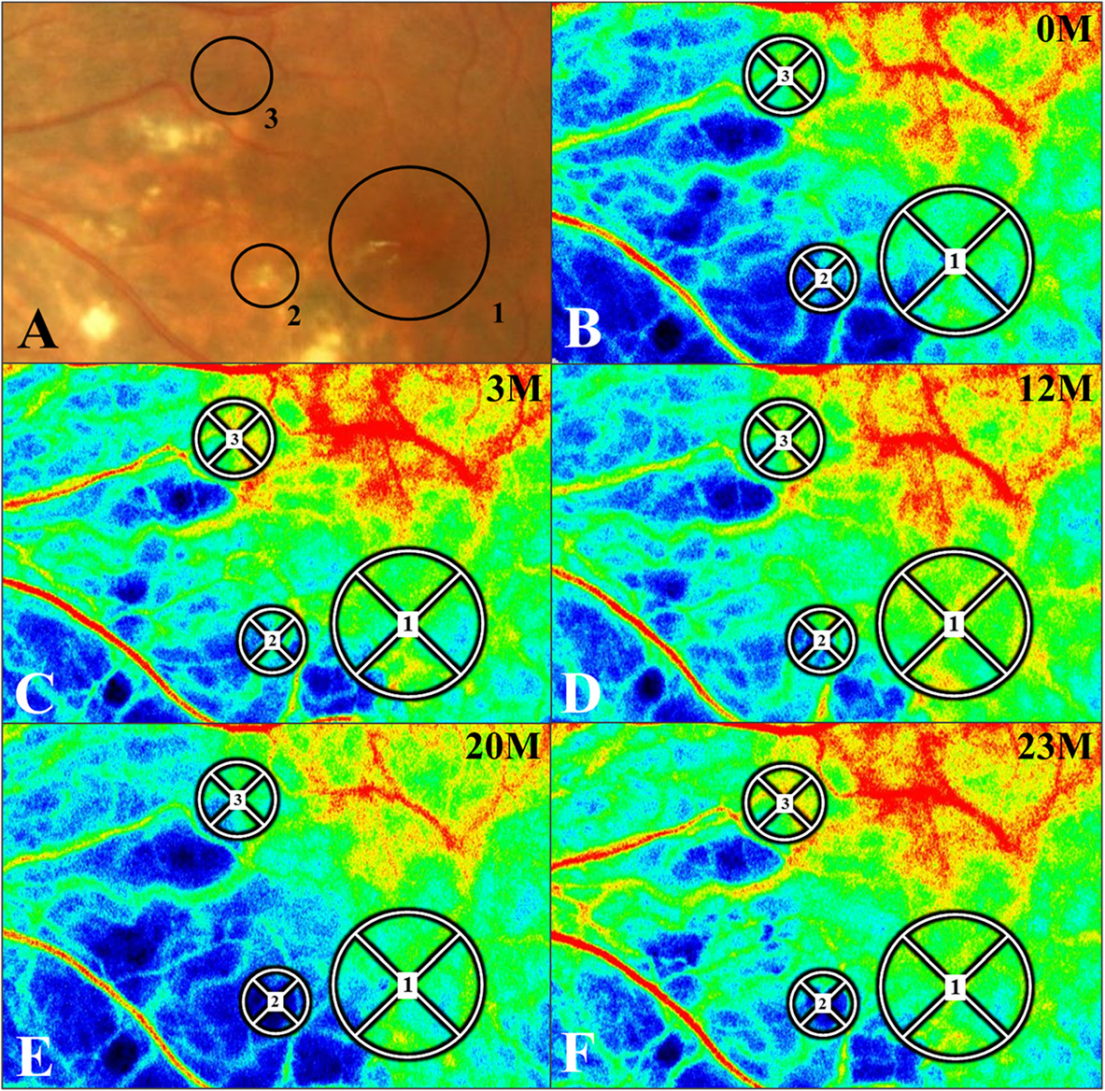
**Changes in the laser speckle flowgraphy (LSFG) color map for the left eye with PIC (B-E). A**, **B**, Three circles were created on the LSFG color map at the initial visit **(B)**, based on the fundus photography at the initial visit (**A**, identical to Figure 1A). Circles 1–3 were located on the fovea, the lesion site and the normal-appearing retinal site, respectively. Red indicates high MBR and blue indicates low MBR. **C**, **D**, In the LSFG color maps obtained 3 and 12 months after systemic prednisolone therapy, MBR had increased at each circle compared to pretreatment values. **E**, **F**, LSFG color map at the time of PIC recurrence (20 months after the initial visit). MBR was lower at this point as compared to 12 months after treatment at each circle **(D)** and increased again after retreatment (23 months after the initial visit).

In order to quantitatively examine choroidal blood flow velocity in the present case of PIC, LSFG measurements using LSFG-NAVI (Softcare, Fukuoka, Japan) were obtained for the eye with PIC and the fellow eye ten consecutive times before treatment, as well as at 1, 3 and 12 months after the initiation of oral prednisolone therapy. The current study was approved by the ethics committee of Hokkaido University Hospital. Informed consent was obtained after an explanation of the nature and possible consequences of the study, which followed the standard of care outlined in the Declaration of Helsinki. The pupils were dilated with 0.5% tropicamide and 0.5% phenylephrine hydrochloride 20 min prior to LSFG testing. During each measurement, eye movement and focus adjustment were monitored using live-capture images. To evaluate the change in relative blood flow velocity at various retinal sites in the PIC eye, measurement circles were set as follows: Circle 1 at the fovea; Circle 2 at a lesion site with exudate; and Circle 3 at a normal-appearing retinal site (Figure 3A). A single circle was set at the center of the macula in the fellow eye. The positions of circles were determined manually by comparing the fundus photographs and the LSFG color map images. The mean blur rate (MBR), a quantitative index of relative blood flow velocity, was calculated in each circle using LSFG Analyzer software (v 3.0.47; Softcare) that automatically set each circle at the same site where a circle had been set at baseline during follow-up. To evaluate changes in average MBR, the rate of change in average MBR vs. initial baseline value was utilized, as previously described [11,12]. This approach was possible with the use of MBR as a quantitative index of “relative” blood flow velocity.

When LSFG measurements are taken for patients with poor visual acuity, the reproducibility of MBR values decreases due to poor visual fixation. In addition, when a lesion such as a subretinal hematoma or bullous retinal detachment blocks laser beam radiation, precise measurements can be difficult to obtain. We assessed the present case with PIC as suitable for LSFG measurements, because this patient maintained good visual acuity during the follow-up without any pathology that would block LSFG measurements.

### Hemodynamics

Within a certain range, the relationship between choroidal blood flow and ocular perfusion pressure (OPP) is bilinear in healthy subjects with normal eyes, as previously demonstrated [13]. To exclude the possibility of such physiological responses from the results, the patient’s blood pressure and intraocular pressure (IOP) were measured to calculate OPP. Mean blood pressure (BPm) was calculated from systolic blood pressure (BPs) and diastolic blood pressure (BPd), according to the following equation: BPm = BPd + 1/3(BPs-BPd). OPP was calculated using the following equation: OPP = 2/3 BPm-IOP [14].

## Results

### LSFG data

**Figure 4:**
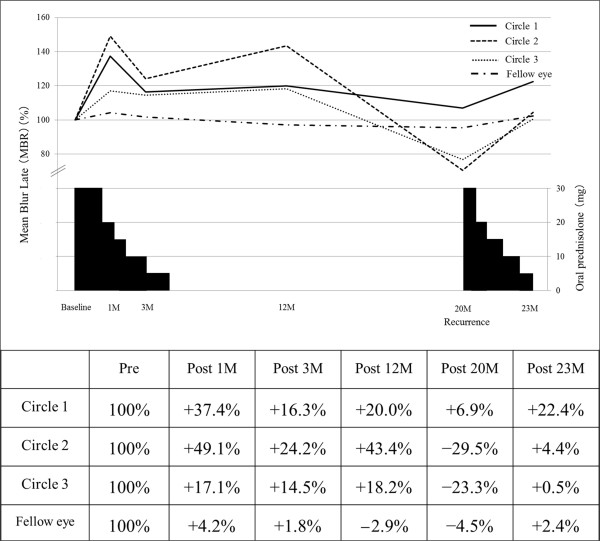
**Trends in MBR in PIC eyes and the fellow eye after the systemic administration of corticosteroids.** In the PIC eye, MBR increased at each circle, sequentially, after the initiation of systemic corticosteroid administration. Although corticosteroid treatment was discontinued after 4 months, MBR remained increased by 20.0%, 43.3% and 18.2%, respectively, at 12 months after treatment compared with baseline measurements (100%). At the time of PIC recurrence (20 months after the initial visit), however, the MBR values in each circle showed decreases of 13-73% compared to the 12-month values. Three months after the resumption of systemic corticosteroid treatment (23 months after baseline), 20-30% increases in MBR compared to the values at 20 months were noted in all circles. However, MBR at the macula in the fellow eye remained unchanged or showed little change (−4.5 ± 4.2%) during follow-up.

The changes in a composite pseudo-color map and the mean MBR during oral corticosteroid treatment at each circle are shown in Figures 3 and 4. The color map demonstrated that MBR increased within the circles 12 months after systemic corticosteroid therapy (Figure 3B-D). Importantly, MBR was shown to decrease at the recurrence of PIC (Figure 3E) and to increase again after retreatment (Figure 3F). The average MBR values were 8.7 ± 0.58, 11.95 ± 0.86, 10.12 ± 0.60, 10.44 ± 1.56, 9.30 ± 1.18 and 10.65 ± 0.65 in Circle 1; 4.95 ± 0.43, 7.38 ± 0.67, 6.15 ± 0.44, 7.10 ± 1.04, 3.49 ± 0.49 and 5.15 ± 0.39 in Circle 2; and 10.83 ± 0.61, 12.68 ± 0.72, 11.32 ± 0.55, 12.08 ± 1.35, 8.31 ± 1.16 and 10.88 ± 0.57 in Circle 3 before treatment and at 1, 3, 12, 20, and 23 months after treatment, respectively. The MBR at the macula of the fellow eye was 10.2 ± 0.56, 10.63 ± 0.53, 10.38 ± 0.32, 9.90 ± 0.59, 9.74 ± 0.54 and 10.44 ± 1.12 before treatment and at 1, 3, 12, 20 and 23 months after treatment, respectively. When the changing rates of macular flow were compared with the mean MBR levels before treatment (100%), 37.4%, 16.3%, 20.0%, 6.9% and 22.4% increases were detected in Circle 1 (the fovea) at 1, 3, 12, 20, and 23 months after treatment, respectively (Figure 4). Similarly, in Circle 2 (the lesion site), 49.1%, 24.2%, 43.4%, −29.5% and 4.4% increases were also noted at 1, 3, 12, 20 and 23 months after treatment, respectively. In Circle 3 (normal-appearing area apart from the lesion), 17.1%, 14.5%, 18.2%, −23.3% and 0.5% increases were measured at 1, 3, 12, 20 and 23 months after treatment, respectively. In contrast, MBR in the fellow eye (the macula) showed little or no change with 4.2%, 1.8%, −2.9%, −4.5% and 2.4% increases at 1, 3, 12, 20 and 23 months, respectively (Figure 4).

### OPP data

OPP was unaltered throughout the course of the disease, with values of 31.0, 30.1, 29.1, 30.9, 30.0 and 32.0 mmHg measured before treatment and at 1, 3, 12, 20 and 23 months after treatment, respectively. OPP in the fellow eye was 30.0, 33.0, 28.4, 29.8, 31.0 and 30.0 mmHg before treatment, and at 1, 3, 12, 20 and 23 months after treatment, respectively.

### Enhanced depth imaging (EDI)-OCT findings

EDI-OCT was performed at the time of PIC recurrence in the left eye (20 and 23 months after the initial visit). The OCT image of the area presented in Figure 1E showed a hump-shaped hyperreflective chorioretinal nodule corresponding to the recurrent PIC lesion (Figure 2D, arrowhead), with surrounding areas showing a loss of IS/OS integrity (Figure 2D, arrows). Average thickness of the choroid in the area surrounding the lesion was measured by 3 authors (K.H., Y.H. and M.S.) as 164.0 ± 0.8 μm. Three months after retreatment, the size of the nodule exhibited no change with slightly increased hyperreflectivity within the lesion (Figure 2E, arrowhead) and the recovery of IS/OS integrity. At the lesion site, choroidal thickness decreased to 122.7 ± 1.9 μm.

## Discussion

In the present study, we examined chronological changes in choroidal blood flow velocity at the macula using LSFG in a patient with PIC. MBR at the lesion site and normal retinal sites surrounding the macula in a PIC eye showed 30–50% increases one month after oral prednisolone therapy, whereas MBR at the macula in the fellow eye increased only 4% during the same period. Although corticosteroid treatment was discontinued after 3 months, the MBR at all sites examined in the PIC eye at 12 months after treatment remained elevated by 20–40% compared to baseline values, along with improvements in visual function and outer retinal morphology. This increase in blood flow was also observed during the course of PIC recurrence, in association with decreased choroidal thickness at the lesion site on EDI-OCT images. To our knowledge, this is the first report in which choroidal circulation hemodynamics was measured quantitatively in a patient with PIC.

Measurements of MBR at the fovea appear to reflect mainly choroidal blood flow velocity because there are no large retinal vessels at the fovea. In the present patient, OPP after systemic corticosteroid therapy remained unchanged compared to the pretreatment value. Therefore, changes in MBR at the macula during follow-up in the PIC eye were due to changes in the velocity of choroidal blood flow but not the systemic circulation. At the time of PIC recurrence, moreover, MBR decreased by 73% compared to the value recorded 12 months after the first treatment. This value had increased by 30% again three months after retreatment. Twelve months after the first treatment, it remains unknown whether MBR returned to physiologically normal levels before the onset of PIC, because LSFG was not performed prior to the initial onset of PIC. However, changes in the MBR, including those observed at the time of recurrence, suggest that choroidal blood flow velocity at the lesion area decreased at the acute phase of PIC but then increased as the associated lesions healed.

Recently, SD-OCT revealed RPE elevation with hyperreflective sub-RPE nodules (Stage II) corresponding to active PIC lesions and subsequently invading the photoreceptors through the RPE breakthrough (chorioretinal nodules, Stage III) [6,7]. Therefore, active PIC lesions appear to affect at least the inner choroid. The wavelength used in laser Doppler flowmetry (670 nm) [15], which is shorter than that used in LSFG (830 nm), predominantly detects red blood cells moving in the choriocapillaris rather than the large choroidal vessels behind it [14]. Accordingly, our data suggest that the choroidal circulatory impairment in PIC extends not only to the inner choroid but also to the deeper layer of the choroid. Furthermore, there were changes detected in the MBR and mfERG responses corresponding not only to the sites of PIC lesions but to normal-appearing retinal sites as well. These results suggest that choroidal circulatory disturbance extended over an area in the posterior pole wider than the PIC lesion sites per se, which is consistent with our recent data on acute macular neuroretinopathy [12].

The mechanism causing choroidal circulation impairment in PIC remains largely unknown. In this study, choroidal thickness decreased and MBR increased concurrently with regression of recurrent PIC, suggesting that choroidal thickness had increased at the active phase, while MBR values had decreased in comparison with those at the remission phase. Recently, another study also demonstrated increased choroidal thickness throughout the posterior pole and/or lesion sites in 30% of eyes with acute PIC [7]. These results suggest that choroidal blood flow decreases together with increases in choroidal thickness during the acute stage of PIC. In an investigation of Vogt–Koyanagi–Harada (VKH) disease, which involves choroiditis predominantly affecting the stroma, previous EDI-OCT [16] and LSFG [17] data demonstrated that macular choroidal blood flow velocity decreased along with increases in choroidal thickness during the acute stage of this disease. Notably, systemic corticosteroid therapy reversed these trends. Furthermore, in acute zonal occult outer retinopathy (AZOOR) and acute macular neuroretinopathy, in association with the AZOOR complex as well as PIC, choroidal blood flow velocity at the lesion site decreases during the acute stage of the diseases [12,18]. In contrast, both blood flow velocity and choroidal thickness increase in the acute stage of central serous chorioretinopathy (CSC) [11,19], which involves a non-inflammatory (i.e., sympathetic or adrenergic) cause. Thus, investigating the changes in MBR and choroidal thickness over the course of diseases may help elucidate their pathogenesis. The LSFG and EDI-OCT results obtained for our PIC patient were comparable to previous observations in VKH disease and showed an “inflammatory pattern” in the choroid. Therefore, our results suggest that inflammation is involved in the pathogenesis of choroidal circulation impairment observed in PIC eyes. Systemic corticosteroid therapy may lead to the improvement of circulation disturbance following resolution of inflammation in the choroid.

In this case, there is a possibility that corticosteroid itself exerted some influence on choroidal circulation besides the MBR changes due to the resolution of PIC. MBR at the macula had increased approximately 5.0% at 1 month after corticosteroid pulse therapy in patients with thyroid-associated ophthalmopathy that developed no retinal or choroidal findings [18]. Because this value of 5% is similar to the MBR value observed in the PIC patient’s fellow eye at the same phase, these results suggest that the influence of corticosteroids on MBR is slight but significant. In our case with PIC, however, an increase in MBR of approximately 20% was maintained even at 12 months after treatment was initiated (prednisolone was tapered up to 4 months). Conversely, there was little change in average MBR in the fellow eye over the same time period. Therefore, it is reasonable to think that the increases in MBR observed in this case stemmed largely from the amelioration of PIC-associated choroidal circulation disturbance.

It would not be fully elucidated whether choroidal thickness changes may affect MBR values, although the effect of choroidal thickening on MBR is estimated to be minimal or negligible compared to the extent of the MBR changing ratio [20]. The MBR represents the velocity of erythrocytes [9,10]. Actually, the MBR changes seen in CSC and VKH disease, both of which involve increased choroidal thickness together with serous retinal detachment (SRD) [16,18], are in the opposite direction (decrease in CSC vs. increase in VKH disease) during regression of the SRD [11,17]. We consider that choroidal thickening develops due to hyperperfusion associated with sympathetic action in CSC [11], whereas choroiditis which is represented as diffuse infiltration of the lymphocytes in the choroidal stroma in VKH disease [17]. Thus, the MBR changes during the course of disease exhibit specific patterns according to the condition’s pathogenesis, independently of the choroidal thickness changes during follow-up. These observations may reduce our attention paid to changes in choroidal thickness for MBR changes.

This study has certain limitations. We present a single PIC patient. One examiner (K.H.) decided the site of each circle for measuring the MBR. These decisions may have been biased because the examiner was not masked to the patient’s diagnosis.

## Conclusions

Macular choroidal blood flow velocity increased and choroidal thickness decreased concurrently with regression of PIC. These results suggest that inflammation-related impairments in choroidal circulation extend beyond the lesion sites and contribute to the pathogenesis of PIC. Further research with a larger number of cases is required to further elucidate the relationship between outer retinal morphology and choroidal circulation in PIC.

## Consent section

Written informed consent was obtained from the patient for publication of this Case report and any accompanying images. A copy of the written consent is available for review by the Editor of this journal.

## Abbreviations

PIC: Punctate inner choroidopathy; LSFG: Laser speckle flowgraphy; mfERG: Multifocal electroretinography; SD-OCT: Spectral-domain optical coherence tomography; BCVA: Best-corrected visual acuity; RPE: Retinal pigment epithelium; FA: Fluorescein angiography; ICGA: Indocyanine green angiography; IS/OS: Photoreceptor inner/outer segment junction; MBR: Mean blur rate; OPP: Ocular perfusion pressure.

## Competing interests

The authors declare that they have no competing interests.

## Authors’ contributions

KH drafted the manuscript, collected the data, and reviewed the literature. WS was involved in the design of the study, interpretation of the data, drafting of the manuscript, and review of the literature. YH and MS participated in the design of the study, collection of the data, interpretation of the data, and review of the literature. SI drafted the manuscript, interpreted the data, and critically reviewed the manuscript. All authors read and approved the final manuscript.

## Pre-publication history

The pre-publication history for this paper can be accessed here:

http://www.biomedcentral.com/1471-2415/14/73/prepub
